# Worry and metacognitions as predictors of the development of anxiety and paranoia

**DOI:** 10.1038/s41598-019-51280-z

**Published:** 2019-10-11

**Authors:** Xiaoqi Sun, Suzanne H. So, Raymond C. K. Chan, Chui-De Chiu, Patrick W. L. Leung

**Affiliations:** 1 Department of Psychology, The Chinese University of Hong Kong, Hong Kong Special Administrative Region, China; 2 Neuropsychology and Applied Cognitive Neuroscience Laboratory, CAS Key Laboratory of Mental Health, Institute of Psychology, Chinese Academy of Sciences, Beijing, China; 3 Department of Psychology, Chinese Academy of Sciences, Beijing, China

**Keywords:** Anxiety, Psychosis, Schizophrenia, Preclinical research, Comorbidities

## Abstract

Recent studies have shown that worry and related negative metacognitions are characteristic in generalized anxiety and paranoia respectively. However, most of these studies did not take into account common co-occurrence of anxiety and paranoia, and longitudinal modelling of the role of worry and metacognitions on the development of anxiety and paranoia is rare. The current study aimed at examining the bidirectional longitudinal relationship between anxiety and paranoia, as well as the importance of worry and metacognitions in the development of these symptoms. Our validated sample consisted of 2291 participants recruited from universities, among whom 1746 participants (76.21%) completed online questionnaires at baseline and at one year, reporting levels of anxiety, paranoia, worry, and negative metacognitions. Structural equation modeling analyses, followed by path comparisons, revealed that anxiety and paranoia mutually reinforced each other over time. Negative metacognitions, rather than worry itself, were contributive to the development of both symptoms over time. Negative metacognitions showed bi-directional relationships with anxiety over the time period assessed and showed uni-directional relationships with paranoia. Clinical implications of our findings are discussed.

## Introduction

Persecutory delusion (PD), an erroneous belief that someone else intends to cause harm to his/herself^1^, is one of the major subtypes of delusions. While PD is characteristic of schizophrenia spectrum disorders^2^, it is also observed in other psychiatric disorders^3^. A wealth of research has shown that persecutory beliefs, ranging from mild paranoia to full-blown PD, can be experienced by individuals without a psychiatric diagnosis, supporting the idea of a psychotic continuum^4,5^. Among individuals who do not have a psychiatric diagnosis, paranoia has been shown to be associated with reduced mental and physical health, and increased behavioral problems (e.g. violence, cannabis use, drinking problem and suicidal ideation)^6–8^. Moreover, presence of delusional ideations predisposes delusion formation^9,10^ and need for care^11^. Therefore, paranoia among non-patients is a topic of interest in its own right, and is important for understanding the development of PD in the clinical population.

Co-occurring anxiety has often been reported by patients who suffer from PD^12,13^ and non-patients experiencing a high level of paranoia^7,14^. According to Freeman and colleagues^15^, paranoia is developed against the backdrop of long-term anxiety. The link from anxiety to paranoia has been supported by clinical^13,16,17^ and non-clinical studies^18–20^. However, it is equally possible that anxiety is a natural response to paranoia as the belief concerns personal threat, and it is a challenge to elucidate on the directionality of association between the two symptoms when both are established among clinical samples. Therefore, the temporal association between paranoia and anxiety needs to be examined among non-clinical individuals, with both symptoms being assessed repeatedly, and both directions of association between considered simultaneously. A recent network analysis study by Kuipers and colleagues suggested that paranoia, as measured by a single item, led to general anxiety after 18 months, but not the other way around^21^. Using more comprehensive measures of paranoia and generalized anxiety, the present study would examine the temporal dynamics between the two symptoms in a large community sample.

Although the common co-occurrence of anxiety and paranoia might suggest direct causal relationships between them, it might also be the case that each is linked to a third underlying common factor^15^. In particular, worry, which is a central cognitive feature of anxiety^22,23^, has recently been theorized as a proximal risk factor for paranoia^24^. Wells and Matthews^25,26^ also proposed that a negative biased style of thinking is a common factor that underlies psychopathology and is caused by dysfunctional metacognitions. This approach was a grounding for Wells’ metacognitive model of generalized anxiety disorder (GAD), in which negative beliefs about the uncontrollability, danger and meaning of worry initiate worry about worry (i.e. meta-worry), and in turn exacerbate worry and intensify anxiety symptoms^27^.

Among both clinical^13,28,29^ and non-clinical populations^18,30^, a general tendency to worry has been shown to have an impact on paranoia. However, these studies relied on a crude measure of a general tendency to worry only, and did not control for level of anxiety, which makes interpretation of results problematic. It has been shown that increased meta-worry^31–33^ and negative beliefs about worry^34–37^, rather than worry itself, distinguished patients with anxiety disorders from non-clinical worriers, and that negative beliefs about worry predicted subsequent emergence of anxiety^38,39^. However, whether negative metacognitions contribute to both paranoia and anxiety over time remains unclear.

A comprehensive assessment of worry and negative metacognitions would allow us to discern whether the impact on paranoia was attributable to processes that are equally common among non-clinical worriers (i.e. worry itself) or processes that are more central to the psychopathology of anxiety (i.e. negative metacognitions). As opposed to cross-sectional studies, a better design would be to assess levels of paranoia, anxiety, worry and negative metacognitions repeatedly, and then to model their inter-relationships simultaneously. It has been increasingly agreed that psychopathology research will be advanced by elucidating the similarities and differences across psychiatric phenotypes and by identifying potential transdiagnostic features and underpinnings^40–43^. Therefore, the current study was aimed to examine the temporal relationship between paranoia and anxiety, and the transdiagnostic roles of worry and negative metacognitions to the development of these symptoms over time. Identification of specific processes that contribute to paranoia would also inform design of worry and metacognitive interventions for paranoia reduction^44–46^.

The present study adopted a structural equation modeling (SEM) approach of a large sample of non-clinical participants, assessed repeatedly. We hypothesized that, after controlling for auto-correlations, baseline level of anxiety will predict level of paranoia at one year, and baseline level of paranoia will predict level of anxiety at one year. As postulated by the metacognitive model of GAD^27^, we also hypothesized that after controlling for covariances, negative metacognitions, rather than worry itself, will predict changes in anxiety and paranoia over time.

## Results

### Demographics and correlation analyses

The mean age of the current sample was 19.77 (SD = 1.41) at baseline, and the majority of them were female (65.9%). Clinical characteristics of participants are summarized in Table 1. Correlations among anxiety, paranoia and each indicators of worry process at baseline and one year are presented in Table 2. All major variables were significantly correlated within and across time points (*ps* < 0.001).

**Table 1:** Sample characteristics.

	Baseline (N = 2291)		1-year follow-up (N = 1746)	
	Mean	SD	Mean	SD
GAD-7	6.11	4.57	5.49	4.53
GPTS total	54.46	19.23	46.27	17.01
GPTS-Ref	32.92	11.39	26.82	10.59
GPTS-Per	21.54	10.01	19.45	7.82
WDQ relationship	7.03	4.38	6.64	4.31
WDQ confidence	8.05	4.76	7.82	4.75
WDQ future	8.00	4.92	7.72	4.86
WDQ work	7.03	4.32	6.58	4.31
WDQ finance	6.62	5.26	5.79	5.01
AnTI Meta-worry	13.88	4.53	13.47	4.62
MCQ U/D	11.74	3.50	11.37	3.84
MCQ NC	12.07	3.43	11.01	3.60

*Note*: GAD-7 = Generalized Anxiety Disorder Scale; GPTS = Green *et al*. Paranoid Thoughts Scales, Ref = Reference Ideation Subscale, Per = Persecutory Ideation Subscale; WDQ: Worry Domain Questionnaire; AnTI: Anxious Thought Inventory; MCQ: Metacognitions Questionnaire-Short Form, U/D: Negative beliefs about worry concerning uncontrollability and danger, NC: Negative beliefs concerning the need to control one’s own thoughts.

**Table 2:** Inter-correlations among variables (N = 1746).

	2	3	4	5	6	7	8	9	10	11	12	13	14	15	16	17	18	19	20	21	22
1. GAD-7 at baseline	0.43	0.33	0.41	0.50	0.46	0.39	0.31	0.62	0.60	0.47	0.50	0.40	0.33	0.34	0.42	0.36	0.33	0.29	0.49	0.46	0.35
2. GPTS-ref at baseline		0.61	0.42	0.44	0.38	0.33	0.32	0.48	0.41	0.37	0.30	0.55	0.40	0.33	0.35	0.30	0.26	0.23	0.37	0.32	0.29
3. GPTS-per at baseline			0.32	0.29	0.28	0.26	0.29	0.39	0.36	0.34	0.24	0.45	0.53	0.24	0.23	0.21	0.20	0.21	0.33	0.29	0.27
4. WDQ relationship at baseline				0.72	0.59	0.50	0.48	0.55	0.48	0.43	0.27	0.39	0.28	0.53	0.46	0.39	0.36	0.32	0.41	0.36	0.31
5. WDQ confidence at baseline					0.71	0.58	0.47	0.67	0.58	0.49	0.33	0.39	0.23	0.47	0.63	0.48	0.42	0.33	0.49	0.43	0.34
6. WDQ future at baseline						0.63	0.54	0.65	0.55	0.47	0.32	0.34	0.23	0.40	0.49	0.60	0.38	0.36	0.47	0.40	0.34
7. WDQ work at baseline							0.54	0.54	0.48	0.42	0.30	0.30	0.20	0.35	0.41	0.42	0.51	0.34	0.40	0.36	0.32
8. WDQ finance at baseline								0.45	0.39	0.34	0.23	0.29	0.23	0.28	0.31	0.31	0.29	0.52	0.31	0.26	0.24
9. AnTI Meta-worry at baseline									0.77	0.71	0.42	0.45	0.34	0.44	0.52	0.50	0.42	0.35	0.63	0.54	0.48
10. MCQ U/D at baseline										0.77	0.40	0.39	0.31	0.37	0.44	0.41	0.35	0.30	0.54	0.54	0.48
11. MCQ NC at baseline											0.31	0.36	0.30	0.32	0.35	0.35	0.31	0.25	0.48	0.44	0.53
12. GAD-7 at 1 year												0.53	0.38	0.40	0.49	0.45	0.44	0.34	0.62	0.63	0.48
13. GPTS-ref at 1 year													0.70	0.52	0.54	0.45	0.42	0.37	0.57	0.50	0.47
14. GPTS-per at 1 year														0.36	0.33	0.30	0.28	0.28	0.42	0.39	0.38
15. WDQ relationship at 1 year															0.72	0.58	0.54	0.48	0.57	0.50	0.46
16. WDQ confidence at 1 year																0.69	0.61	0.45	0.69	0.60	0.51
17. WDQ future at 1 year																	0.62	0.50	0.65	0.55	0.49
18. WDQ work at 1 year																		0.54	0.57	0.52	0.45
19. WDQ finance at 1 year																			0.45	0.39	0.35
20. AnTI Meta-worry at 1 year																				0.80	0.71
21. MCQ U/D at 1 year																					0.69
22. MCQ NC at1 year																					

*Note*: all significant at 0.01 level; GAD-7 = Generalized Anxiety Disorder Scale; GPTS = Green *et al*. Paranoid Thoughts Scales, Ref = Reference Ideation Subscale, Per = Persecutory Ideation Subscale; WDQ: Worry Domain Questionnaire; AnTI: Anxious Thought Inventory; MCQ: Metacognitions Questionnaire-Short Form, U/D: Negative beliefs about worry concerning uncontrollability and danger, NC: Negative beliefs concerning the need to control one’s own thoughts.

### Longitudinal SEM between anxiety and paranoia

**Figure 1:**
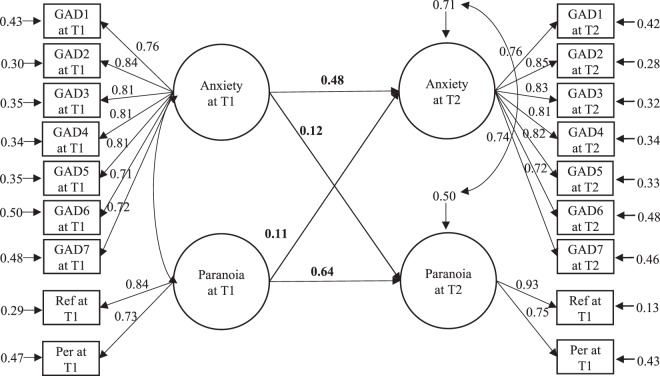
Structural equation model of paranoia and anxiety across time points. *Note*: Values given represent standardized coefficients. T1 = baseline, T2 = 1 year. GAD1-7 = items 1–7 of the Generalized Anxiety Disorder 7-Item Scale; Ref = the reference subscale of the Green *et al*. Paranoid Thought Scales; Per = the persecution subscale of the Green *et al*. Paranoid Thought Scales. All paths are statistically significant (*ps* < 0.01).

Confirmatory factor analysis revealed good model fit (χ^2^ = 891.084, df = 129, *p* < 0.001, CFI = 0.959, SRMR = 0.03, RMSEA = 0.056), with all indicators showing significant and high factor loadings (β = 0.699~0.931, *p* < 0.001). However, factor invariance of “Paranoia” between baseline and one year did not hold (∆χ^2^ = 20.500, ∆df = 1, *p* < 0.001). SEM analysis was performed based on the CFA model with factor loadings of each indicator of “Anxiety” being constrained as invariant across two time points while the factor loadings of two indicators of “Paranoia” being unconstrained. A good model fit was obtained (χ^2^ = 906.187, df = 135, *p* < 0.001, CFI = 0.958, SRMR = 0.031, RMSEA = 0.055). Anxiety at baseline significantly predicted paranoia at 1 year (β = 0.121, *p* < 0.001), and paranoia at baseline also significantly predicted anxiety at 1 year (β = 0.108, *p* = 0.001) (Fig. 1).

Comparison between paths suggested that the standardized regression weight of the path from anxiety at baseline to paranoia at one year and that of the path from paranoia at baseline to anxiety at one year were not significantly different (∆β = 0.013, *p* = 0.46).

### Longitudinal SEM using worry and negative metacognitions as predictors

**Figure 2:**
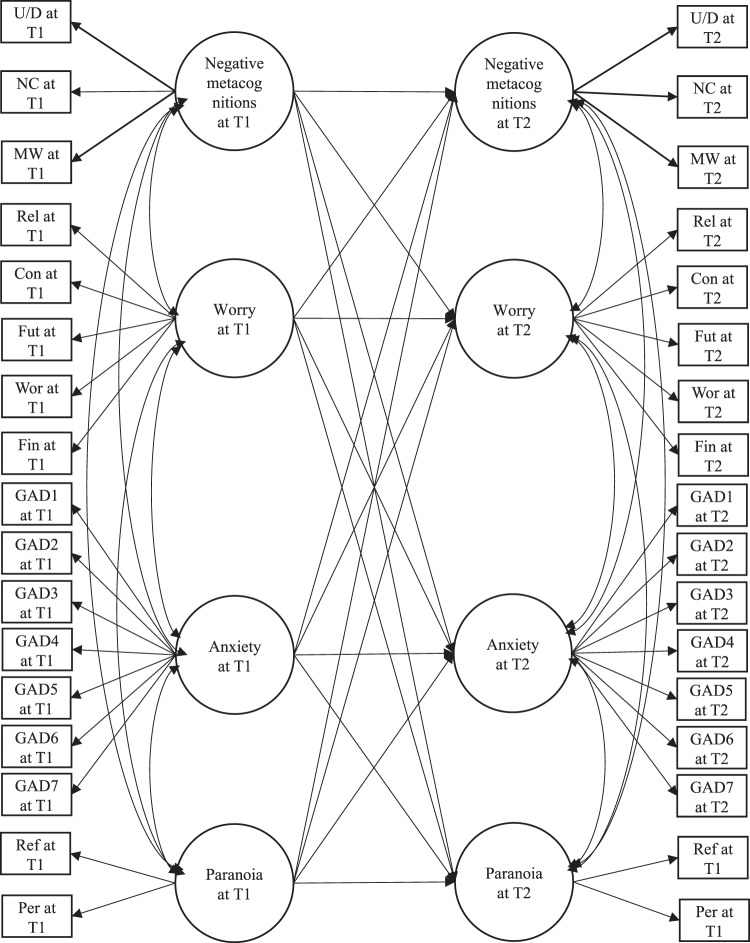
Schematic presentation of the initial structural equation model of relationship among worry, negative metacognitions, paranoia and anxiety across time points. *Note*: T1 = baseline, T2 = 1 year. U/D = the negative beliefs about worry concerning uncontrollability and danger subscale of Metacognitions Questionnaire-Short Form; NC = negative beliefs concerning the need to control worry subscale; MW = the Meta-worry subscale of Anxious Thought Inventory; Rel = the worry about relationship subscale of Worry Domain Questionnaire; Con = lack of confidence subscale; Fut = aimless future subscale; Wor = worry about work subscale; Fin = worry about finance subscale; GAD1-7 = items 1 to 7 of the Generalized Anxiety Disorder 7-Item Scale; Ref = the reference subscale of Green *et al*. Paranoid Thought Scales; Per = the persecution subscale of Green *et al*. Paranoid Thought Scales.

**Figure 3:**
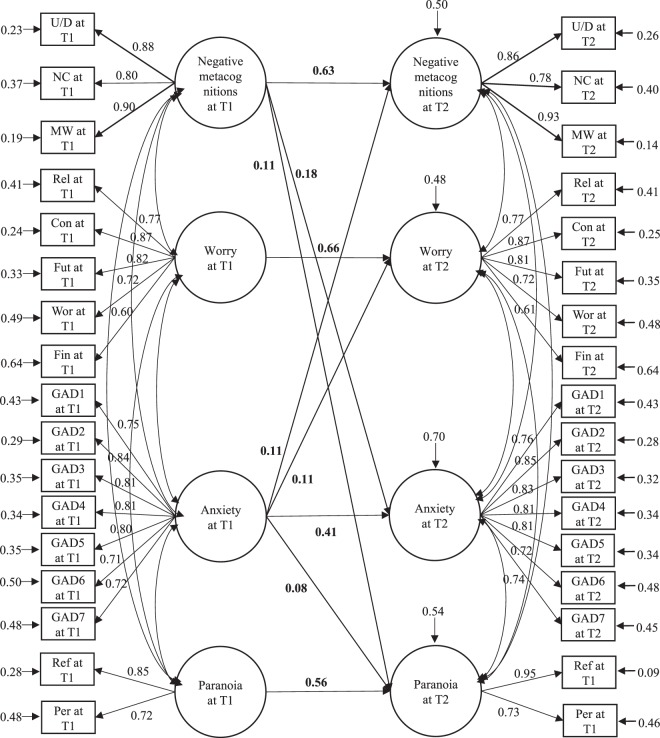
Final structural equation model of relationship among worry, negative metacognitions, paranoia and anxiety across time points. *Note*: Values given represent standardized coefficients. T1 = baseline, T2 = 1 year. U/D = the negative beliefs about worry concerning uncontrollability and danger subscale of Metacognitions Questionnaire-Short Form; NC = negative beliefs concerning the need to control worry subscale; MW = the Meta-worry subscale of Anxious Thought Inventory; Rel = the worry about relationship subscale of Worry Domain Questionnaire; Con = lack of confidence subscale; Fut = aimless future subscale; Wor = worry about work subscale; Fin = worry about finance subscale; GAD1-7 = items 1–7 of the Generalized Anxiety Disorder 7-Item Scale; Ref = the reference subscale of Green *et al*. Paranoid Thought Scales; Per = the persecution subscale of Green *et al*. Paranoid Thought Scales. All paths are statistically significant (*ps* < 0.05).

The CFA model with all latent factors being correlated yielded a good model fit (χ^2^ = 3694.055, df = 499, p < 0.001, CFI = 0.925, SRMR = 0.033, RMSEA = 0.057), with all indicators showing moderate to high factor loadings (β = 0.592~0.953, *p* < 0.001). Latent factors were invariant between baseline and 1 year (*p* > 0.05), except for “Paranoia” (∆χ^2^ = 24.974, ∆df = 1, *p* < 0.001). Therefore, the full SEM model (see Fig. 2 for a schematic diagram) was built upon CFA model with the factor loadings of “Negative metacognitions”, “Worry”, and “Anxiety” being invariant across time while the factor loadings of indicators of “Paranoia” being unconstrained. Although model fit of the initial full SEM model was good (χ^2^ = 3732.372, df = 511, *p* < 0.001, CFI = 0.925, SRMR = 0.034, RMSEA = 0.056, AIC = 114727.313), some paths were not significant. We removed the non-significant paths from the model until all remaining paths were significant. The final model (see Fig. 3) yielded a good model fit (χ^2^ = 3738.970, df = 518, *p *< 0.001, CFI = 0.925, SRMR = 0.034, RMSEA = 0.056). Although it was not significantly different from the initial model according to scaled chi-square test (∆χ^2^ = 8.4964, ∆df = 7, *p* = 0.291), the final model had a lower AIC (114723.408v. 114727.313). Negative metacognitions at baseline significantly predicted paranoia (β = 0.110, *p* < 0.01) and anxiety (β = 0.176, *p* < 0.001) at 1 year. Anxiety at baseline predicted negative metacognitions (β = 0.107, *p* < 0.001), worry (β = 0.105, *p* < 0.001), and paranoia (β = 0.082, *p* = 0.019) at 1 year. However, the predictions of negative metacognition, worry and anxiety at 1 year by baseline paranoia were not significant.

Comparisons between paths suggested that the regression coefficients of the path between negative metacognitions at baseline and anxiety at one year were not significantly different from that of the path leading from negative metacognitions at baseline to paranoia at one year (∆β = 0.051, *p* = 0.302).

The latent interaction between baseline anxiety and negative metacognitions significantly predicted paranoia at one year (B = 0.22, SE = 0.05, *p* < 0.001). Moreover, after adding the interaction term into the model, the path between anxiety at baseline and paranoia at one year became non-significant (*p* = 0.27). Post hoc analysis revealed that for individuals with high levels of negative metacognitions (i.e. 1 SD above the mean), baseline anxiety significantly predicted paranoia at one year (B = 0.16, SE = 0.04, *p* < 0.001), whereas for individuals with low levels of negative metacognitions (i.e. 1 SD below the mean), the link between baseline anxiety and paranoia at one year was not significant (*p* = 0.15). The prediction of anxiety at one year by the latent interaction between baseline paranoia and negative metacognitions was significant (B = −0.08, SE = 0.03, *p* = 0.01). For individuals with low levels of negative metacognitions, paranoia at baseline predicted anxiety at one year significantly (B = 0.08, SE = 0.03, *p* = 0.018), whereas for individuals with high levels of negative metacognitions, the prediction of anxiety at one year by paranoia at baseline was not significant (*p* = 0.76).

## Discussion

The current study was the first attempt to examine longitudinal relationships between paranoia, anxiety, worry and negative metacognitions using a rigorous SEM approach with a large non-clinical sample. We conceptualized paranoia and anxiety as dimensional latent constructs, being indexed with observed symptoms or ideas, and considered cross-lagged relationships between variables using a model comparison approach.

Our results extended the understanding of the longitudinal relationship between paranoia and anxiety. Having controlled for auto-correlations, anxiety predicted change in paranoia, which was consistent with previous longitudinal cohort and experimental studies^8,18–20^. The reverse was also true, so that paranoia and anxiety mutually reinforced each other over one year. However, when worry and negative metacognitions were added to the original SEM model, the paths from paranoia at baseline to anxiety at one year were no longer statistically significant. Together with a recently published cluster analysis study, which found that paranoid individuals tended to be anxious but anxious individuals might not be paranoid^14^, the current finding lends support to the hierarchical structure of paranoia in the general population, which suggests that ideas of persecution build upon more common anxious concerns^5^.

Our study provided evidence for the importance of negative metacognitions in the development of both paranoia and anxiety. After controlling for auto-correlations within variables and cross-lagged associations across variables, negative metacognitions, rather than worry itself, were predictive of both paranoia and anxiety a year after. The magnitudes of predictions of negative metacognitions were comparable for the two symptoms. This is in support of Wells’s metacognitive model, which emphasizes that it is the appraisal of and response to worry that matters in mental ill-health^27^. While a reciprocal relationship between anxiety and negative metacognitions over time was found, the path leading from paranoia to negative metacognitions was not significant. In other words, negative metacognitions and anxiety maintain each other, but such downward spiral is absent in paranoia.

Not only exerting direct influences on both symptoms, our exploratory analyses suggested that negative metacognitions also moderate the interplay between paranoia and anxiety. Anxiety was associated with an increase in paranoia in those scoring high in negative metacognitions only. However, paranoia was associated with subsequent anxiety in those low in negative metacognitions at baseline. The finding that the nature of the temporal relationship between anxiety and paranoia was dependent on level of negative metacognitions is intriguing. It suggests an important role of negative metacognitions in driving the development of paranoid thinking from anxious concerns^5^, and hence more prudent consideration is warranted on theories that make direct links between anxiety and paranoia.

Worry did not emerge as an independent predictor of paranoia when controlling for covariances of negative metacognitions and anxiety, which suggests that the anxiety theory of paranoia should take into account the role of negative metacognitions and further evaluate the relative importance of worry itself and the related metacognitions. In sum, our preliminary results suggest that negative beliefs about worry and meta-worry, rather than worry itself, may be transdiagnostically relevant to anxiety and paranoia. This claim warrants further tests using repeated measures in clinical populations.

Several limitations of current study are noticed. Firstly, we only collected data at two time points, which limited us from investigating the forms of changes over time (e.g. using latent growth curve analysis)^47^, and from examining indirect effects of worry and metacognitions on paranoia via change in anxiety. Secondly, we recruited participants from tertiary institutions. It remains unclear how our results would differ if the sample spans across a wider range of age and education level. Lastly, we appreciate the possibility that development of paranoia and anxiety may also involve other unmeasured mechanisms beyond worry and metacognitions.

## Conclusion

The current findings lend support to the predictive roles of negative metacognitions on changes in both anxiety and paranoia over time. Negative metacognitions showed bi-directional relationships with anxiety over the time period assessed and showed uni-directional relationships with paranoia. Moreover, it appears that negative metacognitions may be important modifiers of the nature of the relationship between the two symptoms, with anxiety being associated with an increase in paranoia among those scoring high in negative metacognitions. Transdiagnostic investigations of the role of metacognitions across psychopathologies will shed light on the use of process-based interventions targeting these processes on treatment for various phenotypes.

## Method

Ethics approval for this study was granted by the Survey and Behavioural Research Ethics Committee (SBREC) of the Chinese University of Hong Kong. All methods were carried out in accordance with relevant guidelines and regulations. Informed consent were obtained from all participants upon recruitment.

### Participants

We included full-time undergraduate students in Hong Kong (age 18–25) and excluded individuals with a current or previous psychiatric diagnosis. Recruitment was carried out using various methods, such as university mass mailing, distribution of leaflets, and promotion on a social media site (i.e. Facebook). At baseline, a total of 2796 participants responded to our survey. We removed 115 participants due to repeated responses and 96 due to invalid responses (not local undergraduate students: n = 19; reporting a current or past psychiatric diagnosis: n = 72; invalid contact information: n = 5). Another 294 participants failed to meet validity criteria (completion time, long-string responses, and odd-even consistency), leaving a final sample of 2291 participants.

All respondents were approached again after one year via e-mails and phone calls. Out of the valid sample of 2291 participants at baseline, 1746 (76.21%) completed the follow-up online survey.

### Measures

Paranoia was measured by the Green *et al*. Paranoid Thought Scales (GPTS)^48^, which consists of 32 items that measure level of reference ideation and level of persecutory ideation over the past month. Good internal consistencies (α = 0.69 to 0.92) and test-retest reliability (r = 0.81 to 0.88) were established^48^. GPTS has been translated into Chinese for the current study (unpublished) and yielded excellent reliability (α = 0.96).

Anxiety was measured by the Generalized Anxiety Disorder 7-Item Scale (GAD-7)^49^. Symptom severity of generalized anxiety was assessed on a 4-point scale over the last two weeks. The Chinese version of GAD-7 yielded good test-retest reliability (α = 0.86) and convergent validity (r = 0.66 to 0.84)^50^. The current sample also reported excellent reliability (α = 0.92).

Worry was measured by the Worry Domain Questionnaire (WDQ)^51^, which is a 25-item questionnaire assessing worries about daily events including relationships, lack of confidence, aimless future, work incompetence and finances. The original WDQ has an acceptable level of internal consistency and good construct validity^51^, and has been translated (with permission) into Chinese for the current study (unpublished). The current sample yielded excellent reliabilities for subscales of WDQ (α = 0.83 to 0.91).

Negative metacognitions were measured by the Anxious Thought Inventory (AnTI)^52^, and the Metacognitions Questionnaire-Short Form (MCQ-30)^53^. AnTI is a 22-item questionnaire comprising social worry, health worry and meta-worry subscales. It has been translated into Chinese for the current study (unpublished). MCQ-30 assesses meta-cognitive beliefs on five dimensions: positive beliefs about worry (PB; “Worrying helps me cope”), negative beliefs about worry concerning uncontrollability and danger (U/D; “Worrying is dangerous for me”), negative beliefs concerning the need to control worry (NC; “I should be in control of my worry all the time”), cognitive confidence (CC), and cognitive self-consciousness (CSC)^53^. For the current purpose, only the meta-worry subscale of AnTI, and the two subscales addressing negative beliefs about worry (i.e. U/D and NC) of MCQ-30 were included. The original versions revealed good internal validities and test-retest reliabilities^52,54^. All three subscales achieved excellent levels of reliability (α = 0.90, 0.83 and 0.78 respectively) in the present study.

### Analysis

Structural equation modeling (SEM) with anxiety, paranoia and worry processes being treated as latent variables was conducted using Mplus version 7^55^. Negative metacognition was indexed with three theory-driven indicators, namely U/D, NC and meta-worry. Worry was indexed with the five subscales of WDQ measuring worry about five distinct domains of life. Paranoia was indexed with two indicators, namely reference ideation and persecutory ideation. Average scores of each subscale were used for all above-mentioned indicators. Anxiety was indexed with the seven items of GAD-7, each capturing a symptom of generalized anxiety.

Correlation analyses between all observed variables were firstly performed. Confirmatory factor analyses (CFA) were then conducted to define the pattern of observed variables for latent constructs and evaluate their factorial invariance between baseline and one year^56^. Full SEM models with uni-directional paths leading from each latent variable at baseline to all latent variables at one year, were carried out subsequently to examine the cross-lagged relationships between latent variables. To test hypothesis 1, a CFA model concerning paranoia and anxiety across time points was tested. This was followed by a SEM model with bidirectional cross-lagged paths between paranoia and anxiety. To test hypothesis 2, models concerning worry, negative metacognitions, paranoia and anxiety were evaluated. Non-significant paths were eliminated to yield a more parsimonious model^57^.

Parameters were estimated by using the robust maximum likelihood (MLR) method^55,58^. Missing values were dealt with full information ML^59^. Model fit was evaluated by chi-square statistic and three goodness-of-fit indices, namely Comparative Fit Index (CFI), Root Mean Square Error of Approximation (RMSEA), and Standardized Root Mean Square Residual (SRMR)^60,61^. Competing models were compared using chi-square difference test with Satorra-Bentler correction^62^, and Akaike’s Information Criterion (AIC) with a lower score indicating better model fit^63^.

As an exploratory analysis, the standardized regression weight of the cross-lagged path leading from anxiety at baseline to paranoia at one year was compared against the path leading from paranoia at baseline to anxiety at one year. Standardized regression weights of paths leading from worry and metacognitions at baseline to paranoia at one year were also compared against corresponding paths leading from that variable at baseline to anxiety at one year. Latent interaction effects between negative metacognitions and each symptom at baseline on the other symptom at one year were explored by using the latent moderated structural equations (LMS) method^64^. Significant results were followed by post hoc analysis using simple slope method^65^.
